# Contemporary Role of Embolization of Solid Organ and Pelvic Injuries in Polytrauma Patients

**DOI:** 10.3389/fsurg.2017.00043

**Published:** 2017-08-07

**Authors:** Nikolaos D. Ptohis, Georgios Charalampopoulos, Adham N. Abou Ali, Efthymios D. Avgerinos, Iliana Mousogianni, Dimitrios Filippiadis, George Karydas, Miltiadis Gravanis, Stamatina Pagoni

**Affiliations:** ^1^Department of Interventional Radiology, General Hospital of Athens “G. Gennimatas”, Athens, Greece; ^2^Second Department of Radiology, General University Hospital “ATTIKON”, Medical School, National and Kapodistrian University of Athens, Athens, Greece; ^3^Division of Vascular Surgery, University of Pittsburgh Medical Center, Pittsburgh, PA, United States; ^4^Third Department of Internal Medicine, General Hospital of Athens “G. Gennimatas”, Athens, Greece

**Keywords:** angiography, embolization, trauma, abdominopelvic, emergency

## Abstract

Abdominopelvic trauma (APT) remains a leading cause of morbidity and mortality in the 15- to 44-year-old age group in the Western World. It can be life-threatening as abdominopelvic organs, specifically those in the retroperitoneal space, can bleed profusely. APT is divided into blunt and penetrating types. While surgery is notably considered as a definitive solution for bleeding control, it is not always the optimum treatment for the stabilization of a polytrauma patient. Over the past decades, there has been a shift toward more sophisticated strategies, such as non-operative management of abdominopelvic vascular trauma for haemodynamically stable patients. Angiographic embolization for bleeding control following blunt and/or penetrating intra- and retroperitoneal injuries has proven to be safe and effective. Embolization can achieve hemostasis and salvage organs without the morbidity of surgery, and the development and refinement of embolization techniques has widened the indications for non-operative treatment in solid organ injury. Moreover, advances in computed tomography provided more efficient scanning times with improved image quality. While surgery is still usually recommended for patients with penetrating injuries, non-operative management can be effectively used as well as an alternative treatment. We review indications, technical considerations, efficacy, and complication rates of angiographic embolization in APT.

## Introduction

Internal abdominal or pelvic bleeding is a significant cause of morbidity and mortality in trauma patients. Mortality rates are particularly high when patients are unstable indicative of exsanguination from solid organ or pelvic injuries (1).

Blunt abdominal trauma is more frequent than penetrating trauma and the most common visceral organ to be affected is spleen followed by liver, while injury of the genitourinary tract accounts for 3–10% of cases (2–4). Pelvic trauma occurs in 3% of bone injuries and pelvic fractures can be associated with abdominal injuries (1, 4, 5). Diagnostic workup in the emergency department must be quick and standardized to avoid delay to bleeding control as time between injury and surgical room is inversely correlated with survival (6). While surgery has been the historical standard of care, recent advances in minimally invasive techniques have revolutionized trauma care. In contemporary practice, the management of a patient with abdominopelvic trauma (APT) is typically carried out by multidisciplinary teams comprised of trauma, vascular and orthopedic surgeons, interventional radiologists, anesthesiologists, and urologists (7).

Interventions in APT focus on hemostasis and life-saving measures aiming at stabilization of the patient; angioembolization has an established valuable role in the management of these injuries (8). We aim to present the contemporary role of embolization of solid abdominal organ and pelvic injuries reviewing indications, technical considerations, efficacy, and complication rates of this technique.

## Computed Tomography (CT) Scan for Assessment of Vascular Injury in the Patient with Abdominopelvic Injury

Ultrasonography has been established as the initial diagnostic approach for patients presenting with blunt APT as it is a rapid and non-invasive imaging modality. Studies have shown that ultrasound is both sensitive and specific in detecting free intraabdominal fluid in adults. However, the non-negligible false negative rates with this modality necessitate subsequent CT scans in most patients (6).

In the past, catheter angiography was used when clinical suspicion for vascular injury was raised. Angiography is an invasive though highly accurate technique that is more time-consuming than a multidetector CT, which is currently the diagnostic modality of choice for the hemodynamically stable patient with APT, assessing bone fractures (with sensitivity and specificity 100%), vascular and solid organ injuries, their grade, and associated complications in a timely fashion (9).

Vascular injury in CT can present with direct signs (i.e., laceration with active hemorrhage, dissection, irregular narrowing, pseudoaneurysm formation, arteriovenous fistula) or indirect signs (i.e., perivascular hematoma, solid organ hypoenhancement) (9).

Vascular injury in APT is more frequent in pelvic fractures and mainly (80–90%) of venous origin and also from bleeding cancellous bone fracture sites, but in unstable pelvic fractures and the hemodynamically unstable patient, arterial bleeding is more frequent and is usually accompanied with a concomitant venous bleed (8, 10, 11).

The most common patterns of arterial injury identified in APT are active hemorrhage depicted as contrast extravasation (blush) and vessel occlusion. CT has a very high (98%) accuracy for identifying blush; however, the absence of contrast extravasation in CT does not always rule out active bleeding (9).

Pelvic hematoma can be easily recognized with CT and a hematoma size >500 cm^3^ is considered highly suspicious for the presence of an arterial injury even in the absence of active contrast extravasation (12).

Multiphasic multidetector CT can differentiate arterial from venous extravasation and current evidence supports the use of CT in APT management to exclude hemorrhage in hemodynamically stable patients (8, 9, 13).

Currently, multi-slice CT (MSCT) scans can image the whole body in less than 30 s. The coupling of MSCT scanners with emergency departments for early trauma management has led to shorter emergency room, operating room, and intensive care unit times (14). Whole-body CT is currently the standard diagnostic modality of choice in hemodynamically stable polytrauma patients.

The contraindication of CT scanning in hemodynamically unstable patients according to Advanced Trauma Life Support recommendations has been questioned by a large retrospective multicenter cohort study, which concluded that there was an increase in survival in hemodynamically stable and unstable patients with APT when whole-body CT was used (15).

## Embolic Agents

A wide variety of embolic agents are available. An ideal embolic agent is one that can be delivered quickly and easily through tortuous arterial anatomy and can reliably lead to hemostasis despite variations in vessel size, type of injury, and patient coagulation status. They can be divided into temporary and permanent agents and furthermore into particulate, mechanical, and liquid agents depending on the material’s structure and mode of action. Embolic agents commonly used in the trauma setting include gelfoam, particulates, coils, plugs, and n-butylcyanoacrylate (NBCA) glue. Operator’s preference, expertise, and the clinical scenario are the main drivers for the various embolic agents used (16).

Particulate agents exist as microspheres, microparticles, and/or sponges. Currently, gelatin sponge or Gelfoam is one of the most commonly used hemostatic agents. This water-insoluble porous complex absorbs an amount of blood 45 times its weight inducing occlusion through close platelet contact within the pores. Its biodegradable and absorbable nature allows it to be used in cases when a temporary and rapid occlusion is needed. Vessels typically recanalize days to weeks later making it the appropriate agent for small vessel injury (17). Gelfoam use, however, requires an intact coagulation cascade and is out of favor in patients with coagulation disorders and/or coagulopathies. It is also associated with infections attributable to air retention (18).

Microparticles such as polyvinyl alcohol (PVA) come in various sizes ranging between 45 and 1,200 µm. Their mode of action is *via* permanent mechanical obstruction as these particles tend to aggregate with each other, even though this same aggregation property can also cause them to occlude the delivery catheter. An advantage with PVA is that embolization with these microparticles is flow directed and, as such, catheter navigation to the target vessel is not necessary (17). However, this does increase the risk of non-target embolization as the microparticles lodge in the distal vasculature.

Coiling is another frequently implemented endovascular intervention that delivers spiral metallic structures that occlude the target vessel through mechanical obstruction. Some coils are covered with synthetic material that promotes coagulation while others have bioactive material that increases the effective coil thickness upon contact with blood. Coils come in various lengths and sizes (loop diameters) as coil sizing is important for successful embolization; undersizing may lead to coil migration while oversizing might cause coil elongation and subsequently inadequate occlusion of the target vessel. Both locations proximal and distal to the bleeding target vessel need to be coiled to prevent back flow from collaterals (17).

Cyanoacrylates have been extensively used in industry and are commercially referred to as “super-glue.” One less toxic form of cyanoacrylates (NBCA) has been developed for medical use. NBCA is a cheap liquid embolization agent ideal for hemorrhagic conditions when rapid target vessel control is required. The embolization is particularly fast that the glue might stick to the catheter upon NBCA delivery necessitating breaking of the catheter tip (17).

Less frequently utilized trauma embolization agents include the Amplatzer vascular plug (AGA medical, Golden Valley, MN, USA) designed to achieve permanent occlusion within 3–5 min. Its main advantage particularly in the trauma setting is the rapidity of agent delivery and the short radiation exposure times.

## Spleen Treatment

The spleen is the most commonly injured intra-abdominal organ, with splenectomy traditionally the mainstay of treatment. Non-operative management for stable patients is widely accepted, especially in younger patients. Reported rates of splenic salvage in stable patients after embolization range from 70 to 97%. Risk factors for failure of non-operative management include age over 55 years, grade IV or V injury, presence of portal hypertension, and CT findings of pseudoaneurysm, vascular lake/blush, and extravasation. Splenic artery embolization is performed for hemodynamically stable patients with grade IV or V injury and in patients with lower-grade injuries with CT findings mentioned above. For lower grade injuries without concerning findings on the initial CT scan, close interval follow-up is performed with embolization considered based on CT findings.

In cases of diffuse splenic injury with multiple areas of bleeding or the “starry-sky pattern,” a proximal embolization technique is employed to decrease the arterial pressure head in the splenic artery to allow the organ time to heal. In cases of focal splenic bleed or pseudoaneurysm formation, superselective embolization may be performed to treat the pseudoaneurysm.

The embolic materials that are used to embolize spleen injuries are coils, gelatin particles, vascular plugs, or PVA particles and the mean technical success is 93.3% and the mean clinical success of non-operative management is 84.6%. Rebleeding rates have a reported overall value of 6.38% (19).

## Liver Treatment

**Figure 1:**
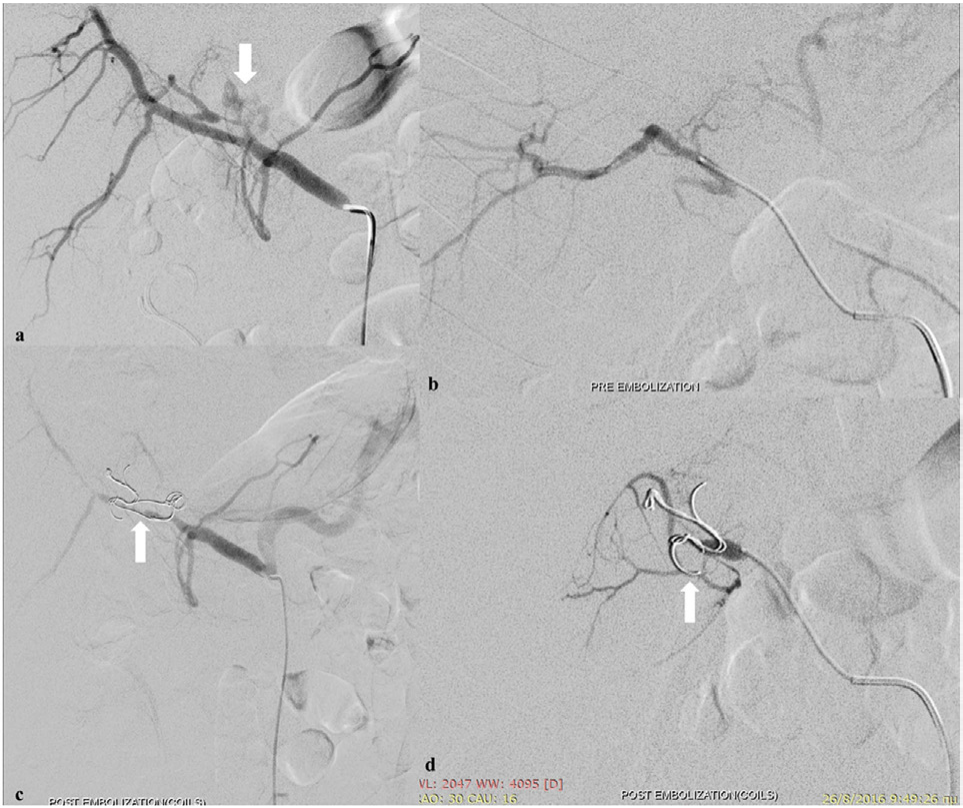
Spasm of the left hepatic artery with pseudoaneurysm presence (arrow) **(A)**. Embolization of the left hepatic artery with 300–500 and 500–700 µm particles (Merit Medical, South Jordan, UT, USA) to occlude the anastomotic branches of the left gastric artery **(B)**. Coil embolization (arrows) (Cook Medical Inc., Bloomington, IN, USA) to occlude the pseudoaneurysm **(C,D)**.

Management for liver injury in unstable patients includes hepatic packing with arterial embolization. Hepatic vein and portal venous injuries are associated with a high mortality and usually necessitate surgical repair even in hemodynamically stable patients. Lower-grade injuries are typically managed non-operatively with angio-embolization indicated for ongoing arterial bleeding or pseudoaneurysm (Figure 1). Hepatic arteries form an extensive network of intrahepatic venous collaterals, which are not apparent angiographically. If a catheter can be advanced across the site of arterial injury, coils may be employed. Otherwise, a liquid or particulate agent is preferred to both occlude the injured vessel and prevent continued extravasation *via* flow from intrahepatic collaterals. High-grade liver injuries by nature involve extensive biliary and vascular disruption. This may be exacerbated by embolization.

The materials that are used to embolize liver injuries are coils, gelfoam, and PVA particles, and the mean technical success is 94.9%, while the mean clinical success approximates 79.8% (19).

## Kidney Treatment

The management of blunt renal injuries is predominantly non-operative in hemodynamically stable patients as the majority of injuries are low grade. However, even in high-grade injuries (according to the American Association for the Surgery of Trauma classification), the non-operative approach is being favored. In a multicenter study, out of 206 patients with grade IV and V kidney injuries, 75% underwent non-operative management with a 92% success rate. 16% of these patients underwent angioembolization (20). Selective angioembolization is being considered as an adjunct to non-operative management in select patients with grade IV and V renal injuries that show evidence of contrast blush on CT scan imaging (21).

Renal arterial injuries are ideally embolized selectively to preserve nephrons. The renal artery and its branches are functionally end arteries without significant collateral supply, thus superselective embolization with micro-coils is the technique of choice for most injuries. For dissection of the main renal artery or proximal branches, a stent may be placed to avoid sacrificing a large territory with coils.

The reported embolic materials that are used in renal injuries are coils, gelfoam, glue, Onyx, and PVA particles (19). Mean technical success is 96.2%, mean clinical success is 90.9%, and overall rebleeding rate can reach 25.6% (19).

## Pelvic Injury

Percutaneous angioembolization is an accepted non-surgical method for treatment of hemorrhage in hemodynamically unstable pelvic fractures, controlling arterial bleeding whereas venous bleeding and hemorrhage from fracture sites, which represent the most frequent pelvic bleeding sites (85%), are effectively controlled by preperitoneal pelvic packing in conjunction with pelvic stabilization (8). Angioembolization for active arterial bleeding in PI has a high success rate (85–100%) and can be repeated in case of ongoing bleeding (11, 22).

Embolization is a time-consuming technique, so careful patient selection is very important in pelvic injury management (6). The depiction of contrast extravasation on CT and the presence of a pelvic hematoma are considered the most important indicators of the need of angioembolization, which may be beneficial regardless of hemodynamic status in the case of arterial contrast extravasation on CT (8). On the other hand, the patient who will need endovascular treatment for PI cannot be predicted by the presence and severity of pelvic fractures (11).

**Figure 2:**
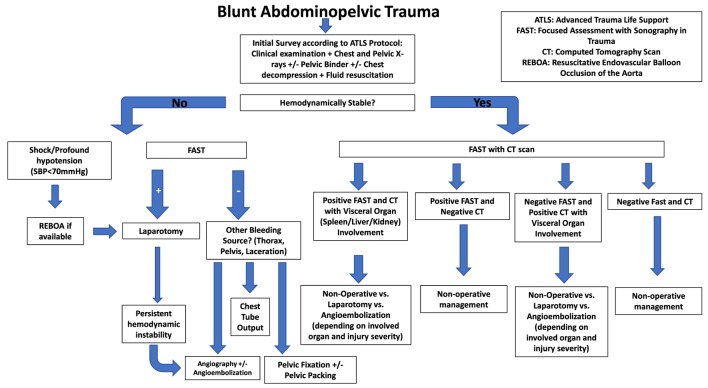
Blunt abdominopelvic trauma management algorithm.

The preferred access site for angiography and embolization is the common right femoral artery and routinely a lower abdominal aorta angiogram is initially performed and then selective pelvic arteriograms are obtained according to the angiographic or CT findings. During angiography, arterial lesions requiring angioembolization are the depiction of contrast extravasation, the presence of a pseudoaneurysm or an arteriovenous fistula or the presence of a cut-off vessel sign (Figure 2) (11).

Technical success is defined as exclusion of the bleeding site at postembolization angiography and has a mean value of 98.9%, while clinical success, which is defined as the percentage of hemodynamically stable patients after angioembolization, has a mean value of 91.7% (19, 23).

In the case of rebleeding, which has a mean rate of 9.7%, angioembolization can be repeated and the reported overall mortality rate, which is defined as death before discharge due to persistent hemorrhage, concomitant trauma, and complications is 15.3% (19).

Although uncommonly utilized, Resuscitative Endovascular Balloon Occlusion of the Aorta (REBOA) has been proposed as a hemorrhage control adjunct (24). A recent multi-institutional study reported a 0.4% use of REBOA in patients presenting with pelvic fractures secondary to blunt trauma with a 2.8% use in pelvic trauma patients presenting with shock (25). However, the exact role and timing of REBOA remains unclear particularly in the context of other hemorrhage control techniques, its unavailability at certain institutions, and the lack of sufficient data supporting its use.

## Complications

Complications of angioembolization can be associated with the arterial access comprising hematoma formation, femoral artery pseudoaneurysm, arteriovenous fistula or dissection, infection, and lower limb ischemia (19, 26). Access site-related complications are not common (3%) and are amenable to non-operative management (26).

Complications can also be related to the embolization procedure such as dissection or rupture of the artery and can also be organ specific such as liver or renal failure, gallbladder ischemia, urinary bladder, gluteal or intestinal necrosis (16, 19, 26). Abscess formation, deep infections, and poor wound healing have also been reported (16, 19, 26).

Systemic complications include major allergic reactions, renal failure, and contrast-induced nephropathy, which is closely related to the dose of iodine per milliliter of glomerular filtration rate and is considerably higher in patients older than 55 years, and in the presence of diabetes mellitus and severe renal insufficiency (26).

The overall complication rate ranges from 8.4 to 11.2% and the majority of complications (83%) can be managed non-operatively by embolization, percutaneous drainage of abscesses, endoscopic retrograde cholangiopancreatography, and hemodialysis (16, 23, 26, 27). It should be emphasized that the true complication rate of angioembolization in APT is not easy to assess as the evidence is not based on prospective randomized controlled trials but on cohort studies; however, the published data suggest that angioembolization is an effective method in controlling life-threatening bleeding (16, 26).

## Conclusion

Angioembolization in APT has an established role aiming to the hemodynamic stabilization of the patient offering high success rates while the majority of angioembolization-related complications can be managed non-operatively. Randomized controlled trials comparing angioembolization with other approaches are very difficult to perform in emergency settings, but current evidence supports that angioembolization is a safe life-saving technique.

## Author Contributions

NP: manuscript editing, overall responsibility. GC: literature research, manuscript preparation. EA, MG, and SP: manuscript editing. IM and DF: literature research, manuscript editing. GK: manuscript preparation. AA: Literature search, manuscript preparation.

## Conflict of Interest Statement

The authors declare that the research was conducted in the absence of any commercial or financial relationships that could be construed as a potential conflict of interest.
